# Modified VMAT Plans for Locally Advanced Centrally Located Non-Small Cell Lung Cancer (NSCLC)

**DOI:** 10.3390/life11101085

**Published:** 2021-10-14

**Authors:** Eva Y. W. Cheung, Virginia H. Y. Kwong, Fandy Y. C. Chan, Dominic Y. T. Cheng, Janice K. Y. Cheng, Sapphire H. Y. Yung, Kiris T. K. Chan, Kelly T. Y. Cheung, Tracy S. W. Cheung, Janna C. L. Yiu

**Affiliations:** 1 School of Medical Health and Sciences, Tung Wah College, 19/F, 31 Wylie Road, Ho Man Tin, Hong Kong, China; 17003980@twc.edu.hk (F.Y.C.C.); 18001627@twc.edu.hk (D.Y.T.C.); 17002895@twc.edu.hk (J.K.Y.C.); 17002595@twc.edu.hk (S.H.Y.Y.); 16003178@twc.edu.hk (K.T.K.C.); 16003318@twc.edu.hk (K.T.Y.C.); 16000196@twc.edu.hk (T.S.W.C.); 15000109@twc.edu.hk (J.C.L.Y.); 2 Department of Clinical Oncology, Prince of Wales Hospital, 30-32 Ngan Shing Street, Shatin, New Territories, Hong Kong, China; Khy806@ha.org.hk

**Keywords:** non-small cell lung cancer (NSCLC), volumetric modulated arc therapy, centrally located, lungs, oesophagus, heart, Durvalumab, immunotherapy, non-PTV lung

## Abstract

Objectives: This study aimed to find the optimal radiotherapy VMAT plans, that achieved high conformity and homogeneity to the planned target volume (PTV), and minimize the dose to nearby organs at risk including the non-PTV lung, heart and oesophagus for patients with centrally located non-small Cell Lung Cancer. Methods: A total of 18 patients who were treated for stage III centrally located non-small Cell Lung Cancer were selected retrospectively for this study. Identical CT datasets, 4D CT and structure dataset were used for radiotherapy planning based on single-planar VMAT (SP-VMAT), dual-planar VMAT (DP-VMAT) and Hybrid VMAT (H-VMAT). For SP-VMAT, one full arc and two half arcs were created on single-plane with couch at 0°. For DP-VMAT, one full arc was created with couch at 0°, and two half arcs with couch rotation of 330° or 30°. For H-VMAT, anterior-posterior opposing fixed beam and two half arcs were planned at couch at 0°. Dose constraints were adhered to the RTOG0617. Dose volumetric parameters were collected for statistical analysis. Results: There were no significant differences for the PTV, HI, CI between the SP-VMAT, DP-VMAT and H-VMAT. For the non-PTV lungs, Dmean, V20, V10, V5, D1500 and D1000 were significantly lower (2.05 Gy, 6.47%, 15.89%, 11.66% 4.17 Gy and 5.47 Gy respectively) in H-VMAT than that of SP-VMAT (all *p* < 0.001). For the oesophagus, Dmax, Dmean, V30 and V18.8 of H-VMAT were 0.08 Gy, 1.73 Gy, 5.54% and 7.17% lower than that of the SP-VMAT plan. For the heart, Dmean, V34, V28, V20 and V10 of DP-VMAT were lower than that of SP-VMAT by 1.45 Gy, 0.65%, 1.74%, 4.8% and 7.11% respectively. Conclusion: The proposed H-VMAT showed more favourable plan quality than the SP-VMAT for centrally located stage III NSCLC, in particular for non-PTV lungs and the oesophagus. It will benefit patients, especially those who planned for immunotherapy (Durvalumab) after standard chemo-irradiation. The proposed DP-VMAT plan showed significant dose reduction to the heart when compared to the H-VMAT plan.

## 1. Introduction

Lung cancer is the second commonly diagnosed cancer in the world, with 2.24 million new cases annually. It has been the leading cause of cancer death, with 1.8 million deaths worldwide [1]. In Hong Kong, the incidence rate is the second (15.4%) among all cancer sites. It has also been ranked as having the highest mortality, of 26.4%, among all cancer sites in 2018 [2].

The standard of care of locally advanced non-small cell lung cancer (NSCLC) is concomitant chemo-irradiation [3]. However, it is known that combining the two treatments increases pulmonary, cardiac and oesophageal toxicities [4,5,6,7], and the median 5-year progression-free survival rate was only 15% [8,9]. The application of immunotherapy (Durvalumab) after chemo-irradiation in the PACIFIC trial demonstrated 17% improvement in the 18-month progression-free survival, but introduced Grade 3 or 4 adverse events, including pneumonitis [10]. Due to the systemic nature of chemotherapy and immunotherapy, the role of radiotherapy (RT) to minimize local regional thoracic toxicity was particularly important. In RT, volumetric modulated arc therapy (VMAT) is adopted in recent years to achieve superior conformity and dose escalation to the tumour, at the expense of more volume of nearby organs receiving a low dose. In this case, minimizing the dose to nearby organs at risk (OARs) becomes crucial when the OAR is radiosensitive. In thoracic regions, the low dose to the lungs, heart and oesophagus are major concerns in radiotherapy planning.

Several studies have demonstrated that modification of co-planar VMAT plans may reduce dose to OARs [11,12,13,14,15,16]. For example, non-coplanar VMAT plans reduced the low dose to the heart during dose delivery, while an ipsilateral lung might expose to higher radiation dose [11]. Hybrid 3DCRT/VMAT (H-VMAT), which includes a pair of AP opposing beams and a pair of partial arcs, is used as a modification of the VMAT plan. It can reduce the dose to the lungs and spinal cord when comparing to co-planar VMAT [16].

The sparing of OARs is challenging when the tumour is centrally located, and/or with lymph node involvement. To deliver the escalated prescribed dose to the target volume, keeping the dose to the lungs, heart and oesophagus less than the tolerance level is also challenging, with the proximity of their location to the target volume. Modified beam angles or arcs entry from different planes can be a solution to disperse the low dose by combining gantry rotation and couch angles appropriately. In this study, through demonstrating different beam and couch configurations, i.e., single-planar VMAT (SP-VMAT), dual-planar VMAT (DP-VMAT) and 3DCRT/VMAT (H-VMAT), and comparing their dose volumetric parameters, it is aimed to explore optimal beam arrangements for stage III centrally located NSCLC patients, to minimize their pulmonary, cardiac and oesophageal toxicities.

## 2. Materials and Methodology

### 2.1. Patient Selection

Patients were selected retrospectively from the Clinical Oncology Department of the Prince of Wales Hospital (PWH), New Territories East Cluster, Hospital Authority, Hong Kong. They were treated for non-small cell lung carcinoma (NSCLC) in 2018–2019. Patients who had been diagnosed with stage III NSCLC, with centrally located tumours in lung, or with mediastinal lymph nodes involvement, whose original radiotherapy treatments were planned on being a free-breathing CT image, were included. Patients who were diagnosed with metastases, received radiotherapy treatment previously or had undergone surgical treatment, were excluded.

Ethics approval was obtained from the Research Ethics Committee of the Hospital Authority, The New Territories East Cluster, Hong Kong SAR (CREC Ref No: CUHK-NTEC 2019.655).

### 2.2. Simulation

Philips Brilliance CT-scanner at PWH was used to perform simulation. Patients were simulated in supine position, with wing board and vacuum vaclok as immobilization devices to support both their arms raised over the head. Slice thickness was set as 3 mm. This is standard setup for lung tumour treatment in PWH. A free-breathing CT image was acquired without a contrast agent. 4D CT was also acquired to measure the tumour motion within the breathing cycle. For this cohort, patients had compromised lung function and large tumours. In consideration of patient tolerance and reproducibility, clinical team decided not to restrict breathing motion using motion management devices. Magnetic resonance image (MRI) was acquired to improve OARs contouring.

### 2.3. Image Registration, Target Volume and OAR Contouring

A total of 18 patients met the above inclusion criteria. Magnetic resonance image (MRI) and free-breathing CT image were co-registered. 4D CT was used to determine the movement of the tumour during the respiratory cycle. The oncologists in PWH were responsible for contouring the planning target volume (PTV). Dosimetrists or radiation therapists in PWH were responsible for contouring the organs-at-risk (OARs), including both lungs, heart, and spinal cord.

The investigators of this study were responsible for contouring the oesophagus with reference to the online RTOG contouring atlas (https://www.rtog.org/CoreLab/ContouringAtlases.aspx, accessed on 1 April 2020): “RTOG1106 Atlas for Organs at Risk (OARs) in Thoracic Radiation Therapy Structures”. All structures were confirmed by radiation therapists in PWH, and approved by certified medical dosimetrist before planning.

### 2.4. Treatment Planning

All treatment plans were done in the radiotherapy planning laboratory at Tung Wah College. To design plans that were compatible to the clinical setting in PWH, the Varian linear accelerator model 21IX (Varian Medical System, Palo Alto, CA, USA) was chosen for all plans in the software Eclipse Radiotherapy Treatment Planning System (Varian Medical Systems, Palo Alto, CA, USA). The version was 15.6. The machine was equipped with 120 High Definition multileaf collimator (MLC) system, and the motion type was sliding window.

For SP-VMAT, DP-VMAT and the VMAT components of H-VMAT plans, 6 MV photon with dose rate of 600 monitor unit (MU) per minute was chosen. Gantry speed was set at 4.8 degree/second. The isocentre and the field sizes were custom fitted to the PTV + 0.5 cm for each patient automatically by the arc geometry tool in the software. To offset the inter-leaf transmission, collimator was rotated 30 degrees in clockwise and counter-clockwise arcs.

### 2.5. Beam and Couch Configuration

**Figure 1:**
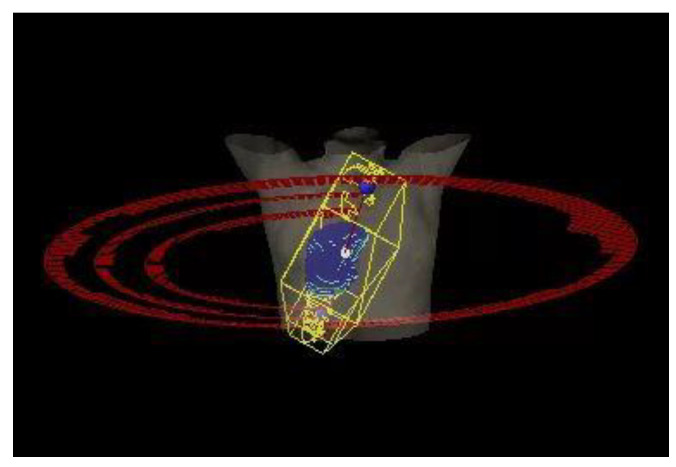
SP-VMAT configuration.

The SP-VMAT plans were composed of three arcs, including one full arc with gantry rotate from 181 degree to 179 degree, and two half arcs with gantry rotate from 181 degree to 0 degree for a tumour situated on right side of the lung; or two half arcs with gantry rotate from 179 degree to 0 degree for a tumour situated on left side of the lung. Couch was set at 0 degrees for all arcs. Details of SP-VMAT plan are shown in Figure 1.

**Figure 2:**
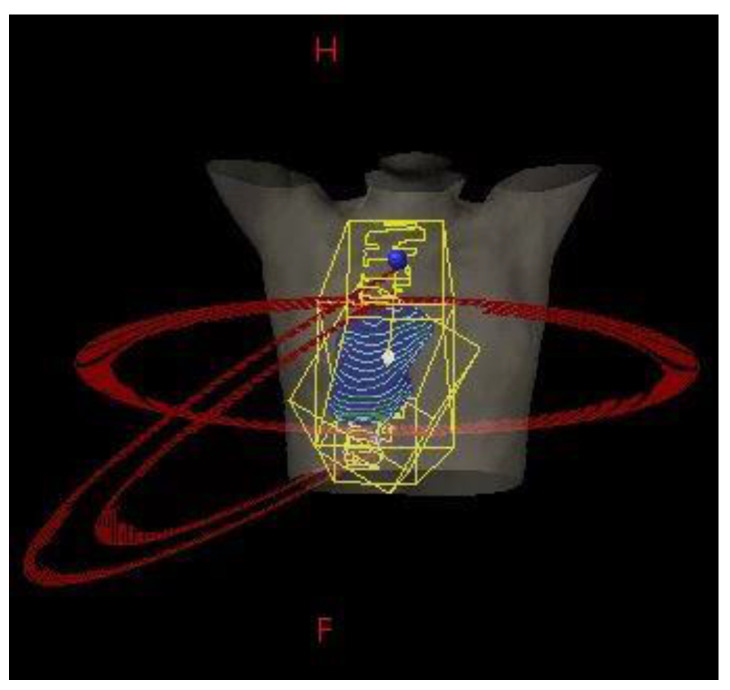
DP-VMAT configuration.

The DP-VMAT plans were composed of three arcs, including one full arc with gantry rotating from 181 degrees to 179 degrees, and two half arcs with gantry rotating from 181 degrees to 0 degrees for a tumour situated on right side of the lung; or two half arcs with gantry rotating from 179 degrees to 0 degrees for a tumour situated on left side of the lung. Couch was set at 0 degrees for the full arc. It was set at 23–30 degrees for the two half arcs for a tumour situated on right side of the lung, and 330–337 degrees for a tumour situated on left side of the lung. Details of the DP-VMAT plan are shown in Figure 2.

**Figure 3:**
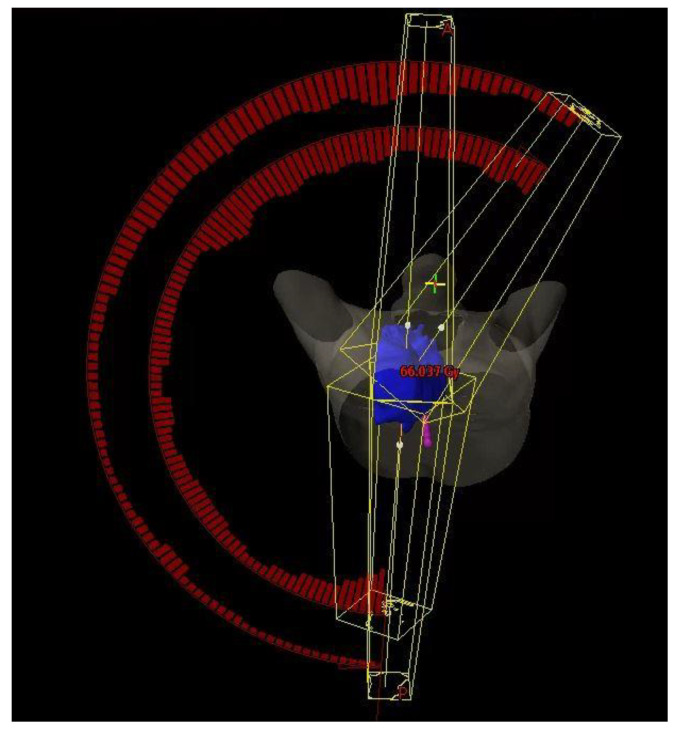
H-VMAT configuration.

The H-VMAT plans were composed of a pair of anterior-posterior opposing (AP) fields (3DCRT component) and two partial arcs (VMAT component). A 6 MV or 10 MV photon was chosen for AP fields to obtain a balanced dose distribution. The field size was determined by the beams-eye-view (BEV) with MLC fitted to the PTV + 0.5 cm margin. The gantry angle of the AP fields was 0 degrees and 180 degrees. The VMAT component was composed of two half arcs, with gantry rotating from 181 degrees to 30 degrees for a tumour situated on right side of the lung; or with gantry rotating from 179 degrees to 330 degrees for a tumour situated on left side of the lung. Couch was set at 0 degrees for all arcs. Details of H-VMAT plan was shown in Figure 3.

### 2.6. Dose Prescription

RTOG 0617 standard dose regime of 60 Gy was adopted [17]. For SP-VMAT and DP-VMAT, dose prescription was 2 Gray (Gy) per fraction (2 Gy/fr), five fractions per week for 30 fractions to a total of 60 Gy. For H-VMAT, 50% of prescribed dose (30 Gy) was allotted to AP fields, which was set as the base plan. The rest of 50% prescribed dose (30 Gy) was allotted to the VMAT for intensity modulation. Altogether, 60 Gy prescribed dose was planned for PTV.

### 2.7. Plan Optimization

The progressive resolution optimiser 3 (PRO3), version 15.6 (Varian Medical systems, Palo Alto, CA, USA) was adopted to optimise the SP-VMAT, DP-VMAT and the VMAT component of H-VMAT plans. Dose constraints are listed in Table 1. During the optimisation process, weighting applied for each constraint was identical in all plans, so as to ensure the DV parameters obtained corresponded to the planning techniques, but not due to the effects of weighting. Details of the inverse planning algorithm constraints and weighting are listed in Table 2. Anisotropic Analytical Algorithm (AAA), Version 15.6, (Varian Medical systems, Palo Alto, CA, USA) was adopted to calculate dose distribution with 1.25 mm grid size after optimisation.

### 2.8. Inter-Planner Variability

To minimize the inter-planner variabilities, planners with similar clinical experience were recruited to perform planning. Each planner was responsible for all three plans, including SP-VMAT, DP-VMAT and H-VMAT for the same patient. Planning procedures were standardized and four sets of CT were used randomly as samples. All planners performed planning on these CT sample sets. Dose-volume metrics of the plans were used to determine the planner’s performance [18]. They started the planning for this study once they met the specified planning goals of each plan.

### 2.9. Evaluation of Treatment Plans

The following criteria were employed as planning goals of each plan:

1. 100% of prescribed dose (60 Gy) should cover more than 98% of PTV
2. Maximum dose of a 2% of PTV should be lower than 108% of the prescribed dose (64.8 Gy).
3. Maximum point dose (i.e., hotspots outside the PTV) should be lower than 108% of the prescribed dose (64.8 Gy).
4. Dose constraints in Table 1 and Table 2 should be met.

To evaluate the plan quality, homogeneity index and conformity index were calculated as follows:

Homogeneity index was calculated as:

HI = (D_2%_ − D_98%_)/DP (1)

where D_2%_ is the dose received by 2% (volume) of planning target volume (PTV), D_98%_ is the dose received by 98% of PTV and DP is the prescribed dose (60 Gy) [19].

Conformity Index was calculated as:

CI = TV/PTV(2)

where TV is the treated volume enclosed by 100% isodose level. PTV is the planned target volume. The CI approach to 1 indicated the dose coverage of target volume conformed more to the PTV [20].

For the PTVs, the maximum dose (D_max_), minimum dose (D_min_), mean dose (D_mean_), minimum dose for 98% volume of PTV (D_98%_), D_95%_, D_50%_ and D_2%_ were calculated.

For non-PTV lung, ipsilateral lung, contralateral lung and both lungs, D_mean_, V_20%_, V_10%_, V_5%_, D_1500cc_, D_1000cc_ were calculated. For the heart, D_max_ and D_mean,_ V_60%_, V_45%_, V_40%_, V_20%_, V_10%_, D_0.1cc_, D_2%_ were calculated. For oesophagus, D_max_ and D_mean_, V_30%_, V_18.8%_ were calculated. For spinal cord, D_max_ and D_0.1cc_ were calculated.

### 2.10. Statistical Analysis

IBM Statistical Product and Service Solutions (SPSS) (Version 25.0) was employed to perform statistical analysis. Non-parametric Wilcoxon signed-rank test was performed to compare the mean of the each dosimetric parameters in SP-VMAT, DP-VMAT and H-VMAT. In this study, *p* values of less than 0.05 were regarded as statistically significant [21].

Certified medical dosimetrists checked and approved all 54 plans to ensure they were clinically acceptable. The results presented in this study were based on these approved plans.

## 3. Result

### 3.1. Patient Demographic

A total of 18 patients were recruited in this study. Their age ranged from 56 to 84 years old. There were 15 males and three females. All patients were diagnosed with NSCLC, 10 were diagnosed with IIIA, and eight were diagnosed with IIIB. The gross tumour size ranged from 97.8 cm^3^ to 823.5 cm^3^, with mean tumour size of 428.76 cm^3^.

### 3.2. Dose-Volumetric of PTV

**Figure 4:**
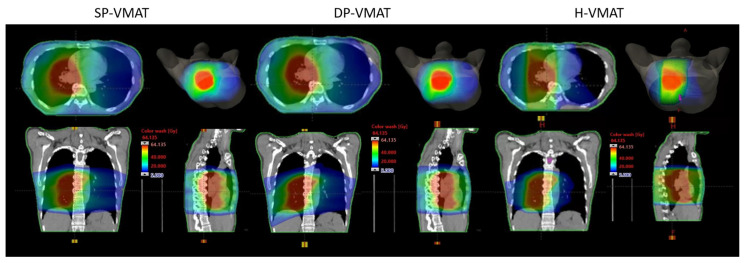
The dose distribution in SP-VMAT, DP-VMAT and H-VMAT in the representative case (case number 8).

The overall HI, CI and dose-volumetric parameters of PTV in SP-VMAT, DP-VMAT and H-VMAT were similar and there were no significant differences. Details are shown in Table 3. The overall dose distributions are shown in Figure 4.

### 3.3. Dose-Volumetric Parameters of the Lungs

For non-PTV lungs, the V5 Gy was significantly higher in DP-VMAT when compared to SP-VMAT. All non-PTV lung parameters in H-VMAT were significantly lower than those in SP-VMAT. For ipsilateral lung, all parameters were significantly higher in DP-VMAT when compared to SP-VMAT, but the difference was minimal. For contralateral lung, all parameters were significantly lower in DP-VMAT when compared to SP-VMAT. Consider H-VMAT plans, all parameters were significantly lower in SP-VMAT in both ipsilateral and contralateral lungs. Details of dose-volumetric parameters of the lungs are shown in Table 4.

### 3.4. Dose-Volumetric Parameters of Centrally Located OARs

DP-VMAT had a similar sparing effect on oesophagus and spinal cord as in SP-VMAT. In DP-VMAT, the heart mean dose was 1.45 Gy significantly lower than that of SP-VMAT (*p* < 0.001). In view of H-VMAT, oesophagus mean dose, V30 Gy and V18.8 Gy were significantly lower in H-VMAT when compared to SP-VMAT, with 1.73 Gy (*p* = 0.02); 5.54%, (*p* = 0.003) and 7.13%, (*p* = 0.002) respectively. This also applied to heart V20 Gy and V10 Gy, being 3.34% (*p* = 0.02) and 7.94% (*p* = 0.007) lower in H-VMAT than that in SP-VMAT.

However, spinal cord maximum dose and D0.1cc were 5.32 Gy (*p* = 0.002) and 5.83 Gy (*p* = 0.02) higher in H-VMAT when compared to SP-VMAT respectively. There was significant higher heart maximum dose, V45 Gy, V40 Gy, D0.1cc and D2% in H-VMAT when compared to SP-VMAT. Details are listed in Table 5.

## 4. Discussion

### 4.1. Significance of the Study

There were no significant differences in the plan quality and dose volumetric parameters in PTV between DP-VMAT and H-VMAT. However, more favourable sparing effects to OARs were found when DP-VMAT and H-VMAT were compared to SP-VMAT.

In H-VMAT, the dose volumetric parameters of lungs (non-PTV lungs, ipsilateral and contralateral lungs) oesophagus and low dose volume of heart (V20 Gy and V10 Gy) were less than those of SP-VMAT, indicated that the sparing effect of these OARs was better when compared to SP-VMAT. For DP-VMAT, there was significant dose reduction to contralateral lung and heart V20 and V10 when compared to SP-VMAT.

### 4.2. Lungs Sparing

Although intensity modulation plans can deliver more homogenous and conformal doses to PTV, when it is applied to lung tumour radiotherapy, large non-PTV lung volume under low dose exposure is a major concern. It is challenging to reduce the non-PTV lung dose when the tumour is centrally located and/or with lymph node involvements. With respect to the clinical trials results of Phase III RTOG 0617, controlling V20 less than 35% can minimize the risk of pneumonitis [17]. Wijsman et al., 2017 suggested that both acute and late pulmonary toxicity were low in both IMRT and VMAT. However, Shi et al., 2010 study suggested that the severe acute radiation pneumonitis incidence with V10 more than 50% and V10 less than 50% were 5.7% and 29.2% respectively (*p* < 0.01) [22]. Shaikh el al 2016 studied 139 patients followed up for 5 years. They suggested that V5, V10, V20 and V30 were positively associated with the risk of grade 2 radiation pneumonitis. V5 less than 65% and V20 less than 25% were identified as threshold of grade 2 radiation pneumonitis [23]. With the application of immunotherapy (Durvalumab) after chemo-irradiation, the constraints were even tightened, with V5 less than 55%, V20 less than 23% and mean lung dose less than 14.8 Gy [24]. In this study, among all lung DV parameters, the non-PTV lung was optimized based on the dose constraints only. The ipsilateral lung, contralateral lung and both lungs may have DV parameters over the dose constraints. In DP-VMAT, dose delivered to contralateral lung was significantly reduced in V10, V5, D1500 and D1000 when compared to SP-VMAT. However, the dose delivered to ipsilateral lung was increased. When considering non-PTV lungs, V5 was significantly increased by 3.45% (*p* = 0.006), while there was no significant difference in other parameters. The obtained result was expected. For centrally located tumours, both SP-VMAT and DP-VMAT deposited a low dose to ipsilateral and contralateral lung, to achieve a high dose to the PTV. The volume of lung irradiated with a low dose should remain the same in both plans.

In contrast, for H-VMAT, there was significant dose reduction in all DV parameters to non-PTV lung, both lungs, ipsilateral lung and contralateral lungs. In non-PTV lungs, the reduction in volume of V20, V10 and V5 was 6.47%, 15.89% and 11.66% respectively (*p* < 0.001) when compared to SP-VMAT. The reduction of volume was even more for a contralateral lung, with 6.09%, 27.27% and 22.14% in V20, V10 and V5 respectively. Comparing our H-VMAT results with the abovementioned studies, the probability of grade 2 pneumonitis was much reduced without compromising the dose delivered to PTV [16,21,22]. The effect could be obvious for patients who planned to receive immunotherapy after chemo-irradiation. The non-PTV lung dose of our H-VMAT resulted in a V5 of 49.29% ± 9.51; V20 of 21.83% ± 4.26 and mean lung dose of 13.19 Gy ± 2.47, which was lower than the threshold that patients developed pneumonitis at in Landman et al.’s 2021 study [24]. Thus, by controlling the non-PTV lung dose, the progression-free survival would be improved by the application of immunotherapy while limiting the risk of developing pneumonitis.

The major contributor for the V20, V10 and V5 volume reduction was the AP opposing beam arrangement that delivered 50% of the prescribed dose, the VMAT provided intensity modulation to improve the homogeneity and conformity to the PTV by the rest of 50% prescribed dose. Hence, the non-PTV lung volume irradiated by low dose could be reduced significantly. Our results are coherent with Mayo et al.’s 2008 study, which illustrated that the hybrid of static and intensity modulated beams could better spare the OARs [25]. The benefit was noticeable for contralateral lungs, when a partial arc could be employed to replace the full arc. While in the SP-VMAT and DP-VMAT application, the full arc could not be replaced by partial arc to maintain PTV full coverage and homogeneity.

### 4.3. Heart Sparing

Cardiac injury is a major radiation induced side effect of Hodgkin lymphoma [26,27] and breast cancer [28,29] that developed 10 years after receiving radiotherapy treatment. These patients are young and have favourable prognosis. However, the prognosis for patients with stage III NSCLC is poor, with median survival less than 2 years. In addition, stage III NSCLC patients are generally older and may have more vascular comorbidities and smoking history. The development of cardiac toxicities may be shortened. Wang et al., 2017 reported that patients with mean heart dose received over 20 Gy had a 21% to develop cardiac events in 2 years [30]. Allan et al., 2015 reported that 8.4% and 13% of patients developed cardiac events in 9 and 24 months respectively after they received approximately 63 Gy. Although no association between heart dose and survival has been reported, they suggested to limit the heart V50 and V60 to reduce the rate of cardiac events [31]. All VMAT plans proposed in this study showed very low dose to the heart, even though the heart was near to the PTV in some cases. V20 and V10 had significant reduction in DP-VMAT and H-VMAT when compared to SP-VMAT, suggesting that DP-VMAT and H-VMAT had a better heart sparing effect.

### 4.4. Oesophagus Sparing

Radiation induced oesophagitis has been reported where mean dose, V30 and V60 are the predictor with threshold of 27.5 Gy, 43% and 12.4%, with V60 is the best among all [16,32]. In our study, V60 was less than 1% in all three plans, where mean dose and V30 were under the threshold suggested. In particular, significant reduction of mean dose and V30 by 1.73 Gy and 5.54% was found in H-VMAT when compared to SP-VMAT, suggesting that H-VMAT had good sparing effect on oesophagus.

### 4.5. Low Dose Irradiation to Normal Body Tissue

Although the risk of developing post-radiation sarcoma is rare with a 0.06% incidence rate, it is associated to the exposure of normal body tissue [33]. Our results demonstrated that the V10 and V30 of normal body tissue received 7.34% and 2.78% lower dose in H-VMAT than that in SP-VMAT. Our finding was coherent with Kim et al., 2020 study, that low dose received by normal body tissue could be reduced by including arcs from different planes [11].

### 4.6. Duration of Treatment

For advanced stage NSCLC patients, patient tolerance and treatment reproducibility are our major concerns [16]. With a similar plan quality as IMRT, VMAT is getting popular for its short treatment time. For an SP-VMAT plan, the average treatment time is 4 min to deliver 2 Gy in three arcs. One couch kick is required for DP-VMAT, an additional minute is required to move the couch manually; or 20 s is required to move the couch by dynamic rotation. For H-VMAT, two static fields are included which can be controlled by an automatic field sequencer, and an additional 2 min is needed when compared to SP-VMAT. In this case, the delivery time remains short in DP-VMAT and H-VMAT. The treatment accuracy and reproducibility can be further improved when breathing motion control could not be offered in our setting.

### 4.7. Patient Safety

For radiotherapy treatment deliver to the thorax, collision is one of the concerns when treatment is delivered by arcs from different planes. The probability of collision increases with couch rotation of more than 30 degrees, large patient size and off-centre tumour location [34]. In this study, no couch rotation is employed in SP-VMAT and H-VMAT. For DP-VMAT, 23–30 degree of couch rotation was employed to ensure clearance. In clinical setting, the pre-defined trajectories and immobilization devices must be checked carefully before DP-VMAT plan optimization. Treatment setup trial and moving the couch manually during the first treatment are recommended for DP-VMAT [11].

For DP-VMAT in this study, only a single couch rotation was proposed due to the patient positioning. Full arc delivery from opposite couch rotation was restricted, as the patient was supine with their arms raised over head. For future studies, a partial anterior arc with gantry of 45 to 315 degrees can be adopted with an additional couch kick at opposite direction. It may help to improve the dose reduction in ipsilateral lung and the coverage of PTV if the tumour was located superiorly.

### 4.8. Limitations of the Study

The sample size of was small in this study. This was mainly due to the eligibility criteria of the disease. To minimize the small sample size effect, we used identical CT sets from all patients we collected to produce the SP-VMAT, DP-VMAT and H-VMAT plans to demonstrate the dose volumetric difference was due to the technique difference, but not other factors.

In this study, the Varian 21IX linear accelerator was selected based on the real clinical setting in PWH. It was not equipped with jaw tracking. Previous studies demonstrated that jaw tracking technique reduced the dose to an ipsilateral lung VMAT in locally advanced NSCLC without jeopardising the conformity and homogeneity to PTV. In addition, there was significant dose reduction of V20 in contralateral lung, V35 in the oesophagus and other OARs [35,36]. With the jaw tracking capability, radiation dose to OARs might be further reduced in SP-VMAT, DP-VMAT and H-VMAT. Further investigation is suggested in future study.

For DP-VMAT, ±23 to 30 degree couch rotation was chosen in the current study to demonstrate the effect on OARs sparing. Other couch and gantry configurations can be adopted in future studies to evaluate which settings could achieve better OARs sparing effects for patients with NSCLC.

About the Weighting of 3DCRT/VMAT in H-VMAT plan, 50%:50% of prescribed dose was allocated in 3DCRT and VMAT components. Other combinations including 40%:60%; 60%:40% and 30%:70% were planned for the selected case as a trial for plan quality evaluation. Mayo et al. reported that using a two-thirds dose in static fields and a one-third dose in intensity modulated beams improved OARs sparing capabilities [25]. Our findings were comparable with the results from Chan et al. in 2011 that two partial arcs plus two static fields with 50% in each of the component yielded a better conformity to PTV and reduced the dose to non-PTV lung [37]. Other combinations showed either less reduction in volume to non-PTV lungs (less weighting in 3DCRT, i.e., 40%:60% and 30%:70%), reduced in conformity to PTV (30%:70%) or higher dose to the heart (60%:40%). Based on the trial findings, the optimal weighting of 50%:50% was chosen for the H-VMAT plan in this study.

Other than modifying treatment plans (from SP to DP or H-VMAT) before treatment starts, adaptive radiotherapy is an approach which focuses on re-planning during treatment course, to improve the therapeutic ratio and minimise the dose to OARs [38]. The application was essential for patients with substantial tumour shrinkage and geometrical uncertainties during the treatment course [39]. The dose reduction demonstrated in this study will be further enhanced by incorporating it with adaptive radiotherapy.

## 5. Conclusions

The proposed DP-VMAT and H-VMAT showed similar HI, CI and PTV dose volumetric, but significant dose reductions to OARs when compared with the SP-VMAT for centrally located stage III NSCLC. The proposed H-VMAT showed favourable plan quality especially to non-PTV lungs and the oesophagus. In particular, the H-VMAT plan could be considered for patients who were planned for immunotherapy after standard treatment. The DP-VMAT plan showed significant dose reduction to the heart when compared to the H-VMAT plan. Therefore, modified VMAT proposed in this study can be considered in a clinical setting, to develop a new standard of care for locally advanced centrally located NSCLC patients that pulmonary, cardiac, and oesophageal functions can be preserved after radiotherapy.

## Figures and Tables

**Table 1:** Planning Dose constraints.

Critical Organ/Organ-at Risk	Dose Constraint
Spinal Cord	Maximum dose < 45 Gy
Non-PTV Lung	Mean dose < 20 Gy V20 < 35% V5 < 65%
Oesophagus	Maximum dose < 63 Gy (105% of prescribed dose) Mean dose < 34 Gy
Heart	Mean dose < 35 Gy V40 < 80% V45 < 60% V60 < 30%

**Table 2:** Inverse planning algorithm constraints.

Structure	Dose Constraint	Weight
PTV	Maximum dose < 60 Gy Minimum dose > 57 Gy	100 100
Spinal Cord	Maximum dose < 45 Gy	100
Non-PTV lung	Mean dose < 20 Gy V20 < 35%	50
Heart	Mean dose < 35 Gy	50
Oesophagus	Maximum dose <63 Gy	20

**Table 3:** Dose-volumetric parameters of the PTV.

PTV DV Parameters	SP-VMAT	DP-VMAT	H-VMAT	(DP-VMAT Minus SP-VMAT)		(H-VMAT Minus SP-VMAT)	
	Mean ± S.D.	Mean ± S.D.	Mean ± S.D.	Diff	*p*	Diff	*p*
Maximum Dose (Gy)	64.59 ± 0.67	64.68 ± 0.81	65.54 ± 0.57	0.09	0.472	0.95	0.23
Mean Dose (Gy)	61.49 ± 0.17	61.45 ± 0.26	61.71 ± 0.26	−0.04	0.446	0.23	0.22
Minimum Dose (Gy)	55.25 ± 1.14	57.15 ± 1.09	54.26 ± 2.08	1.92	0.43	9.01	0.60
D98% (Gy)	60.42 ± 0.34	60.37 ± 0.34	60.54 ± 0.26	−0.05	0.248	0.12	0.33
D95% (Gy)	60.75 ± 0.21	60.69 ± 0.24	60.77 ± 0.27	−0.06	0.267	0.02	0.95
D50% (Gy)	61.51 ± 0.17	61.46 ± 0.28	61.66 ± 0.32	−0.05	0.36	0.16	0.12
D2% (Gy)	62.49 ± 0.244	62.48 ± 0.36	63.25 ± 0.35	−0.01	0.744	0.75	0.26
Homogeneity Index	0.0338 ± 0.006	0.0354 ± 0.01	0.0447 ± 0.01	0.0016	0.215	0.01	0.1
Conformity Index	1.1889 ± 0.09	1.1894 ± 0.07	1.2165 ± 0.089	0.0006	0.943	0.03	0.132

**Table 4:** Dose-volumetric parameters of the lungs.

	DV Parameters	SP-VMAT	DP-VMAT	H-VMAT	DP-VMAT Minus SP-VMAT		H-VMAT Minus SP-VMAT	
		Mean ± S.D.	Mean ± S.D.	Mean ± S.D.	Diff	*p*	Diff	*p*
Non-PTV lung	Mean Dose (Gy)	15.24 ± 3.18	15.53 ± 2.76	13.19 ± 2.47	0.28	0.112	−2.05 *	<0.0001
	V20 Gy (%)	28.3 ± 6.98	28.52 ± 6.97	21.83 ± 4.26	0.22	0.647	−6.47 *	<0.0001
	V10 Gy (%)	51.73 ± 12.05	51.46 ± 9.93	35.84 ± 6.08	−0.26	0.695	−15.89 *	<0.0001
	V5 Gy (%)	57.95 ± 14.65	61.41 ± 11.83	49.29 ± 9.51	3.45 *	0.006	−11.66 *	<0.0001
	D1500 (Gy)	10.44 ± 4.26	10.54 ± 3.92	6.29 ± 2.34	0.097	0.948	−4.17 *	<0.0001
	D1000 (Gy)	16.67 ± 5.34	17.12 ± 4.89	11.19 ± 3.88	0.458	0.349	−5.47 *	<0.0001
Both Lungs	Mean Dose (Gy)	17.31 ± 3.59	17.58 ± 3.16	15.31 ± 2.98	0.27 *	0.002	−2.00	<0.0001
	V20 Gy (%)	31.51 ± 7.36	31.73 ± 7.37	25.23 ± 5.01	0.22	0.231	−6.27 *	<0.0001
	V10 Gy (%)	53.87 ± 12.03	53.64 ± 9.88	38.64 ± 6.65	−0.22 *	0.001	−15.23 *	<0.0001
	V5 Gy (%)	59.34 ± 14.39	62.69 ± 11.54	48.16 ± 9.81	3.34 *	<0.0001	−11.19 *	<0.0001
	D1500 (Gy)	12.17 ± 5.04	12.18 ± 4.78	7.37 ± 3.08	0.02 *	0.002	−4.80 *	<0.0001
	D1000 (Gy)	19.68 ± 7.27	20.39 ± 7.04	14.52 ± 7.12	0.71 *	0.012	−5.16 *	<0.0001
Ipsilateral Lung	Mean Dose (Gy)	24.46 ± 5.55	25.64 ± 5.03	23.88 ± 5.12	1.18 *	0.002	−0.58	0.058
	V20 Gy (%)	52.0 ± 12.75	53.58 ± 11.85	45.72 ± 9.53	1.58	0.231	−6.82 *	<0.0001
	V10 Gy (%)	63.48 ± 14.88	69.81 ± 11.74	59.30 ± 12.11	6.32 *	0.001	−4.18 *	0.002
	V5 Gy (%)	61.99 ± 16.18	69.74 ± 11.80	59.37 ± 16.77	7.75 *	<0.0001	−2.62 *	0.004
	D1500 (Gy)	2.05 ± 2.61	3.06 ± 4.10	1.69 ± 2.09	1.01 *	0.002	−0.36 *	0.003
	D1000 (Gy)	13.23 ± 10.03	15.85 ± 9.07	10.26 ± 8.10	2.61 *	0.012	−2.97 *	0.002
Contralateral Lung	Mean Dose (Gy)	9.41 ± 2.56	8.71 ± 1.72	5.82 ± 1.75	−0.7 *	0.002	−3.59 *	<0.0001
	V20 Gy (%)	8.71 ± 7.54	7.42 ± 5.18	2.62 ± 3.13	−1.29	0.231	−6.09 *	<0.0001
	V10 Gy (%)	42.63 ± 16.45	35.36 ± 11.69	15.37 ± 9.97	−7.27 *	0.001	−27.27 *	<0.0001
	V5 Gy (%)	66.04 ± 14.36	64.65 ± 12.59	43.89 ± 14.38	−1.39	<0.0001	−22.14 *	<0.0001
	D1500 (Gy)	0.78 ± 1.15	0.688 ± 1.01	0.54 ± 0.75	−0.1 *	0.002	−0.24 *	0.017
	D1000 (Gy)	5.08 ± 3.84	4.33 ± 2.60	5.86 ± 13.32	−0.75 *	0.012	0.77 *	0.006

* *p* value < 0.05.

**Table 5:** Dose-volumetric parameters of centrally located OARs.

	DV Parameters	SP-VMAT	DP-VMAT	H-VMAT	DP-VMAT Minus SP-VMAT		H-VMAT Minus SP-VMAT	
		Mean ± S.D.	Mean ± S.D.	Mean ± S.D.	Diff	*p*	Diff	*p*
Oesophagus	Max Dose (Gy)	59.94 ± 9.15	60.16 ± 8.42	59.87 ± 11.42	0.22	0.679	−0.08	0.744
	Mean Dose (Gy)	24.33 ± 6.71	24.54 ± 6.02	22.60 ± 7.44	0.20	0.472	−1.73 *	0.02
	V30 Gy (%)	39.54 ± 13.49	37.93 ± 13.67	33.99 ± 14.17	−1.61	0.193	−5.54 *	0.003
	V18.8 Gy (%)	49.57 ± 13.53	49.03 ± 10.62	42.44 ± 14.80	−0.54	0.647	−7.13 *	0.002
Spinal Cord	Max Dose (Gy)	35.87 ± 7.26	35.3 ± 7.65	41.19 ± 3.83	−0.14	0.679	5.32 *	0.002
	D_0.1cc_ (Gy)	33.42 ± 7.53	33.4 ± 7.52	39.29 ± 3.93	−0.06	0.811	5.83 *	0.02
Heart	Max Dose (Gy)	57.85 ± 16.58	58.87 ± 14.37	62.51 ± 7.56	1.02	0.102	4.67 *	0.002
	Mean Dose (Gy)	13.66 ± 8.66	12.20 ± 8.33	13.74 ± 8.68	−1.45 *	<0.001	0.08	0.879
	V60 Gy (%)	2.15 ± 2.31	2.2 ± 2.39	2.56 ± 2.15	0.05	0.217	0.41	0.079
	V45 Gy (%)	5.472 ± 5.21	5.472 ± 5.1	9.61 ± 8.23	0.00	0.99	4.14 *	0.003
	V40 Gy (%)	7.22 ± 6.56	7.03 ± 6.61	12.88 ± 11.21	−0.19	0.28	5.86 *	0.001
	V20 Gy (%)	26.37 ± 20.63	21.57 ± 19.03	23.03 ± 18.06	−4.8 *	0.001	−3.34 *	0.02
	V10 Gy (%)	47.25 ± 33.39	40.14 ± 30.59	39.3 ± 25.82	−7.11 *	<0.001	−7.94 *	0.007
	D_0.1cc_ (Gy)	56.94 ± 16.80	57.8 ± 14.91	61.48 ± 8.65	0.86	0.094	3.68 *	0.001
	D_2%_ (Gy)	48.8 ± 18.17	48.55 ± 18.66	53.86 ± 14.29	−0.25	0.811	5.31 *	0.002
Body	Mean Dose (Gy)	8.34 ± 2.40	8.41 ± 2.23	7.64 ± 2.11	0.07	0.327	−0.70 *	<0.001
	V10% (%)	32.80 ± 8.36	33.64 ± 7.36	25.46 ± 6.43	0.84 *	0.037	−7.34 *	<0.001
	V30% (%)	16.15 ± 5.13	15.30 ± 4.97	13.37 ± 3.97	−0.86 *	0.001	−2.78 *	<0.001

* *p* value < 0.05.

## Data Availability

The clinical and MRI data are not publicly available for patient privacy protection purposes.
